# Cost-effectiveness analysis of treatment timing considering the future entry of lower-cost generics for hepatitis C

**DOI:** 10.2147/CEOR.S171248

**Published:** 2018-09-20

**Authors:** Katherine Heath

**Affiliations:** 1 Mathematical Ecology Research Group, Department of Zoology, University of Oxford, Oxford OX1 3PS, UK, katherine.heath@new.ox.ac.uk; 2 New College, Oxford OX1 3BN, UK, katherine.heath@new.ox.ac.uk

**Keywords:** ledipasvir, sofosbuvir, cost-per-QALY-gained, drug patent expiry, HCV, hepatitis C treatment

## Abstract

**Background:**

Cost-benefit analyses are crucial to inform treatment policies, particularly when the cost of patented drugs is very high. The cost of patented drugs is the limiting factor in hepatitis C treatment. However, hepatitis C drug costs are expected to fall following patent expiration, due to generic drug introduction.

**Methods:**

An existing mathematical model by Shih et al was extended to consider lower-cost future generics in health economic models of hepatitis C. The model compared the cost-effectiveness of treating patients now with patented drugs vs postponing treatment until after patent expiration.

**Results:**

For ledipasvir-sofosbuvir, this study finds that it is almost always more cost effective to treat hepatitis C with high-cost patented drugs immediately rather than waiting for patent expiry. For ledipasvir-sofosbuvir, a generic would need to enter the market at <16.40% of the patented price for delayed treatment to be cost effective. The further that patent expiry is in the future, the more cost effective delayed treatment becomes; however, uncertainty about generic pricing and market entry times are also higher if patent expiry is in the distant future.

**Conclusion:**

It is more cost effective to treat hepatitis C sooner rather than later, regardless of the stage of the disease, and despite the high cost of patented drugs. However, patented drugs are being produced globally for prices much lower than those seen in the UK. Therefore, negotiation of patented drug prices with pharmaceutical companies may be a crucial step in cost effective treatment of hepatitis C.

## Introduction

Generic drugs are chemical compounds which are therapeutically equivalent to their brand name counterparts. Generic drugs enter a pharmaceutical market when the patent for the brand version expires. Studies have found that generic drugs can enter the market at 60% of the brand price.^1^,^2^ This can decline to less than 40% two years after market entry.^3^ Comparative economic models of drug cost-effectiveness often fail to consider the implications of lower-cost future generics.^4^,^5^ This study will consider how acknowledgment of lower-cost future generics in economic models may influence treatment decisions, with emphasis upon infectious diseases such as hepatitis C, which do not require lifetime treatment.

A key determinant of health technology funding is the incremental cost-effectiveness ratio (ICER). In the UK, the National Institute for Health and Care Excellence (NICE) imposes an ICER threshold range of £20,000–£30,000 per quality-adjusted life year (QALY) gained, which has not changed in NICE guidelines for over 15 years.^6^ In 2005, Shih et al^4^ proposed a mathematical framework allowing cost-effectiveness analyses to incorporate the effects of future generics on ICER estimates. In 2015, this framework was further considered by Guertin et al.^5^ Both Shih et al^4^ and Guertin et al^5^ concluded that failure to consider the future introduction of lower-cost generics could lead to biased ICER estimates when comparing two drugs. However, to the best of the author’s knowledge, it has not been questioned whether the options of treating patients using a high-price branded drug in the present vs treating with a lower-cost generic in the future could be incorporated into cost-effectiveness analyses and what the consequent effect would be on the ICER. The motivation for asking this question is what the survival benefit would need to be to justify funding early treatment in cases where patented drugs are very costly.

The cost of patented drugs is a limiting factor in hepatitis C treatment, although highly effective drugs are now available. In 2011, protease inhibitors such as telapravir and boceprevir were introduced, which increased the efficacy of therapy for genotype one in Western countries. In 2014, sofosbuvir, simeprevir, daclatasvir, and ombitasvir were released. Crucially, these drugs have exhibited sustained virological response (SVR) rates close to 100% in addition to reduced adverse event rates, wider genotype efficacy, and reduced treatment duration.^7^–^11^ However, the cost of sofosbuvir alone is priced at USD $84,000 per 12-week treatment course.^12^

Due to high drug costs, the UK alone treats only 3% of infected patients.^13^ Little progress has been made in low/middle income countries as the market is less lucrative.^14^ To ease the financial burden, high income countries prioritize patients based on their health state. However, a meta-analysis by Simmons et al^15^ has underlined the importance of treating all patients, regardless of their health state, by concluding that the achievement of an SVR is viable, even in high-risk populations where hepatitis C transmission is greatest.

Current pricing significantly reduces access to new, highly efficacious therapy, preventing the realization of hepatitis C eradication goals. Therefore, proper economic evaluation in terms of the QALYs gained for treating individuals in the present using high-cost patented drugs as opposed to waiting for patent expiry is important.

This study investigates the costs and benefits associated with (a) treating now using the high-cost patented drug and (b) postponing treatment until release of the lower-cost generic drugs with reference to the ICER.

## Methods

Both Shih et al^4^ and Guertin et al^5^ consider the cost-effectiveness of two treatments: a new treatment and a comparator. This study adapts Shih et al’s^4^ mathematical framework to compare two situations in relation to a single treatment for which a lower-cost generic becomes available at some point in the future. All analyses were conducted in R Statistical Software version 3.3.2.^16^

### Shih et al’s (2005) original framework

Shih et al’s^4^ original framework will first be presented before revisions are outlined. Let *T*=the final time point in the model’s time horizon; *C*_1_(*t*)=the mean total cost associated with the new drug at time *t; C*_0_(*t*)=the mean total cost associated with the comparator drug at time *t; ND*_1_(*t*)=the mean non-drug costs of the new drug at time *t; ND*_0_(*t*)=the mean non-drug costs of the comparator drug at time *t; P*_1_(*t*)=the price of the new drug at time *t; P*_0_(*t*)= the price of the comparator drug at time *t; Q*_1_(*t*)=the mean total quantity of the new drug consumed at time *t; Q*_0_(*t*)=the mean total quantity of the comparator drug used at time *t; E*_1_(*t*)=the mean effectiveness of the new drug at time *t; E*_0_(*t*)=the mean effectiveness of the comparator drug at time *t;* r=discount rate; *j*=1 if new drug or 0 if the comparator drug; *Δ*C=incremental cost when ignoring the future introduction of lower-cost generic versions; *ΔC*’=incremental cost when considering the introduction of lower-cost generic versions; *Δ*E=incremental effectiveness when ignoring future introduction of lower-cost generic versions; *ΔE*’=incremental effectiveness when considering future introduction of lower-cost generic versions; ICER=ICER when ignoring the future introduction of lower-cost generic versions; and ICER”=ICER when considering the future introduction of lower-cost generic versions.

Ignoring the future introduction of lower-cost generic versions, the real prices of the new and comparator drugs will stay constant over time (ie, *P_j_*(*t*)=*P_j_*). Therefore, when the future introduction of lower-cost generic versions is ignored:

$$\Delta C=\sum_{t=0}^{T}\frac{P_{1}(t)\cdot Q_{1}(t)-P_{0}(t)\cdot Q_{0}(t)}{{\left(1+r\right)}^{t}}+\sum_{t=0}^{T}\frac{ND_{1}(t)-ND_{0}(t)}{{\left(1+r\right)}^{t}}$$ (1)

$$\Delta E=\sum_{t=0}^{T}\frac{E_{1}(t)-E_{0}(t)}{{\left(1+r\right)}^{t}}$$ (2)

$$ICER=\frac{\Delta C}{\Delta E}$$ (3)

Shih et al^4^ then consider the effects of generic drug entry. If T* is the time of generic drug entry into the market, then *P*_0_*_P_* is the price of the old drug prior to patent expiration and *P*_0_*_G_* is the price of the old drug after generic drug entry. Therefore

$$P_{0}(t)=\left\{ \begin{array}{l} P_{0P}if\;t<T^{*}\\ P_{0G}if\;t\geq T^{*} \end{array}\right.$$ (4)

Assuming that *ΔE*=*ΔE′*, then

$$\begin{array}{l} \text{Δ}C’=\\ \overset{T{*}_{1}-1}{\underset{t=0}{\sum }}\frac{P_{1P}(t)\cdot Q_{1}(t)}{{\left(1+r\right)}^{t}}+\overset{T}{\underset{t=T{*}_{1}}{\sum }}\frac{P_{1G}(t)\cdot Q_{1}(t)}{{\left(1+r\right)}^{t}}-\\ \overset{T{*}_{0}-1}{\underset{t=0}{\sum }}\frac{P_{0P}(t)\cdot Q_{0}(t)}{{\left(1+r\right)}^{t}}-\overset{T}{\underset{t=T{*}_{0}}{\sum }}\frac{P_{0G}(t)\cdot Q_{0}(t)}{{\left(1+r\right)}^{t}}+\\ \overset{T}{\underset{t=0}{\sum }}\frac{ND_{1}(t)\cdot ND_{0}(t)}{{\left(1+r\right)}^{t}} \end{array}$$ (5)

### Modified framework: extension of parameter *Q* to incorporate population dynamics

**Figure 1:**
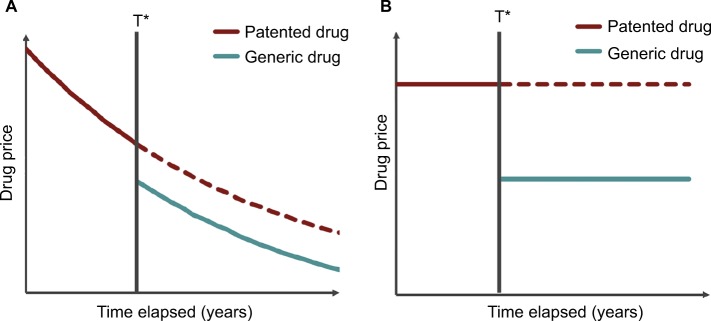
Example of the dynamics of (**A**) discounted and (**B**) undiscounted prices of a patented drug and its generic alternative before and after patent expiry. T* is the time of patent expiry and generic drug entry into the market.

The current study modified the above framework used by Shih et al^4^ and Guertin et al^5^ to consider a single drug and its future generic as opposed to evaluating a new drug and a comparator. A graphical representation of the scenario considered by extension of Shih et al’s^4^ mathematical framework can be observed in Figure 1.

For this single drug, *P_P_* is the cost per unit of the patented drug; *P_G_* is the cost per unit of the lower-cost future generic; *Q_P_* is the quantity of the patented drug used; *Q_G_* is the quantity of the generic drug used; and T* remains the time of patent expiry. T* is also the time of generic entry into the market, assuming that the generic becomes available as soon as the patent expires. The costs incurred at a time, *t*, were calculated as

$$C(t)=\frac{P_{P}(t)\cdot Q_{P}(t)}{{\left(1+r\right)}^{t}}+\frac{P_{G}(t)\cdot Q_{G}(t)}{{\left(1+r\right)}^{t}}$$ (6)

and the total costs incurred for a specific set of parameters were calculated as

$$C=\sum_{t=0}^{T^{*}-1}\frac{P_{P}(t)\cdot Q_{P}(t)}{{\left(1+r\right)}^{t}}+\sum_{t=T^{*}}^{T}\frac{P_{G}(t)\cdot Q_{G}(t)}{{\left(1+r\right)}^{t}}$$ (7)

assuming that the patented drug ceases to be used once the generic is available. The *Q* terms, *Q_P_* and *Q_G_*, were expanded in order to model a changing population of infected and cured individuals. Additional parameters were introduced: *I*=the number of infected individuals; *R*=the number of cured individuals; *D*=the number of dead individuals (with *D_I_* and *D_R_* being the number who die from infection and natural causes, respectively); *a_P_*=the coverage rate of the patented drug; *a_G_*=the coverage rate of the generic drug. The quantity of each drug used was defined by

$$\begin{array}{l} Q_{P}(t)=\{\begin{array}{ll} I(t)\cdot \alpha_{P} & if\;t<T^{*}\\ 0 & if\;t\geq T^{*} \end{array}\\ Q_{G}(t)=\{\begin{array}{ll} 0 & if\;t<T^{*}\\ I(t)\cdot \alpha_{G} & if\;t\geq T^{*} \end{array} \end{array}$$ (8)

The population was assumed to be closed. Two different mortality rates were applied. The first, *μ_R_*, was the natural mortality rate expected in a non-infected population, which was modeled as the natural mortality rate expected in the UK, increasing with age, taken from the Office for National Statistics.^17^ The second was the mortality rate for infected individuals, *μ_I_. μ_I_* can be set as a function of *μ_R_* depending on the disease of interest.

Parameters *e_P_* and *e_G_* were the efficacies of the patented and future generic drug, respectively. The number of infected individuals of age, *a*, at a time, *t*, was calculated by

$$I_{\left(a,t\right)}=\left\{ \begin{array}{l} \left(I_{\left(a-1,t-1\right)}-\alpha_{P}\cdot e_{P}I_{\left(a-1,t-1\right)}\right)\cdot \left(1-\mu_{I_{\left(a\right)}}\right)\;if\;t<T^{*}\\ \left(I_{\left(a-1,t-1\right)}-\alpha_{G}\cdot e_{G}I_{\left(a-1,t-1\right)}\right)\cdot \left(1-\mu_{I_{\left(a\right)}}\right)\;if\;t\geq T^{*} \end{array}\right.$$ (9)

and the number of recovered individuals of age, *a*, at a time, *t*, was calculated by

$$R_{\left(a,t\right)}=\left\{ \begin{array}{l} \left(R_{\left(a-1,t-1\right)}-\alpha_{P}\cdot e_{P}I_{\left(a-1,t-1\right)}\right)\cdot \left(1-\mu_{R_{\left(a\right)}}\right)\;if\;t<T^{*}\\ \left(R_{\left(a-1,t-1\right)}-\alpha_{G}\cdot e_{G}I_{\left(a-1,t-1\right)}\right)\cdot \left(1-\mu_{I_{\left(a\right)}}\right)\;if\;t\geq T^{*} \end{array}\right.$$ (10)

The total number of individuals who die over the course of the time horizon, *T*, as a result of infection, *D_I_*, was calculated by

$$D_{I}=\sum_{t=0}^{T}D_{I(t)},where\;D_{I(t)}=\sum_{a=0}^{A}\mu_{I_{\left(a\right)}}\cdot I_{\left(a,t\right)}$$ (11)

and the total number of dead individuals was calculated by

$$\begin{array}{l} D=\sum_{\left(t=0\right)}^{T}D_{I(t)}+\sum_{\left(t=0\right)}^{T}D_{R(t)},where\;D_{I(t)}+D_{R(t)}\\ =\sum_{\left(a=0\right)}^{A}(\mu_{I_{\left(a\right)}}\cdot I_{\left(a,t\right)})+\sum_{\left(a=0\right)}^{A}\left(\mu_{R_{\left(a\right)}}\cdot R_{\left(a,t\right)}\right). \end{array}$$ (12)

An age structure was imposed on infected individuals. With each iteration of the model, individuals were assumed to gain 1 year of age and moved from class *I*_(_*_a_*,*_t_*_)_ to class *I*_(_*_a_*_+1_,*_t_*_+1)_.

Using equations (8) and (9) the quantity of patented and generic drugs used was calculated. These were then substituted back into equation (7) to calculate the total cost incurred by treatment over the specified time horizon. The total number of QALYs when treatment is administered (ie, where *a_P_*>0 and *a_G_*>0) was calculated using the utilities assigned to each health state. *ψ_R_* is the utility value for recovered, *ψ_I_* for infected, and *ψ_D_* for dead individuals. The total number of QALYs was, therefore, calculated as:

$$QALYs=\sum_{t=0}^{T}\psi_{I}\cdot I(t)+\sum_{t=0}^{T}\psi_{R}\cdot R(t)+\sum_{t=0}^{T}\psi_{D}\cdot D(t)$$ (13)

#### Cost-effectiveness comparison

This study considered the ICER associated with treating an infectious disease with branded drugs before patent expiry, as opposed to treating them after patent expiry using generic drugs. The incremental cost, *ΔC* is the difference between the total costs of the current treatment regimen, *C_1_*, and the comparator treatment regimen, *C_2_*, where *C_1_* and *C_2_* were calculated using equation (7).

$$\Delta C=C_{2}-C_{1}$$ (14)

The denominator to calculate the ICER was the difference between the total number of QALYs over the time horizon, *T*, of the current treatment regimen, QALY_1_, and the comparator treatment regimen, QALY_2_, where QALY_1_ and QALY_2_ were calculated using equation (13).

$$\Delta QALY=QALY_{2}-QALY_{1}$$ (15)

The ICER was calculated as

$$ICER=\frac{\Delta C}{\Delta QALY}$$ (16)

### Case study: timing of hepatitis C treatment considering future generic drug entry

**Figure 2:**
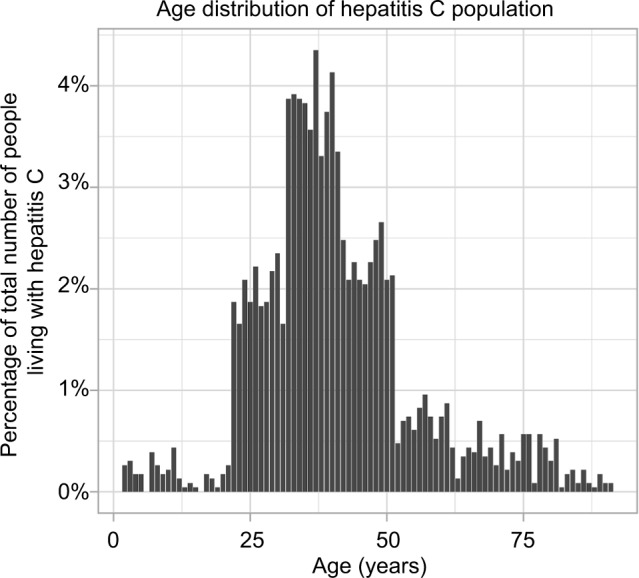
Age distribution of hepatitis C infected individuals used in the model. **Notes:** The total infected population size was 214,000. The distribution was estimated using Mohsen (2001), who reported an age distribution in 10-year intervals in the Trent region.^19^ Each 10-year interval was split randomly to generate 1-year intervals.

The size of the infected population was assumed to be 214,000 at time *t*=0, which is the number of people in the UK thought to be living with chronic hepatitis C.^18^ The age distribution was fitted using a 2001 prospective study by Mohsen^19^ over five centers in the Trent region, specifying the age distribution of hepatitis C patients. Mohsen^19^ described the age distribution of the study cohort in 10-year intervals (for example, birth year 1991–2001), with the number of patients in each interval. In order to generate a distribution with 1-year intervals, the number of patients in each 10-year interval was split into ten parts of random size. The age distribution is displayed in Figure 2. Adaption of the model is possible using age distributions from empirical studies in different geographical locations; similar data were found to exist for studies in Italy and China.^20^,^21^

**Figure 3:**
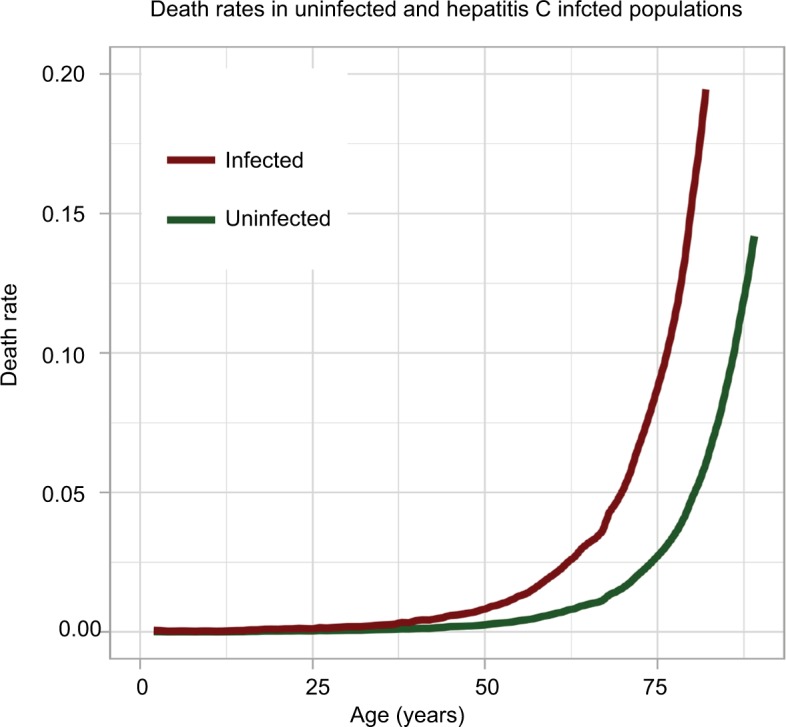
Death rates in uninfected and hepatitis C infected individuals. **Notes:** The uninfected mortality rate is the natural mortality rate expected in the UK, taken from the Office for National Statistics.^17^ The infected mortality rate is calculated by multiplying the uninfected mortality rate by a factor of 3.22.^22^

The hepatitis C mortality rate, *μ_I_*, was assumed to be higher than the natural death rate by a pre-specified factor. This factor was derived from the relevant literature. McCombs et al^22^ observed death rates of 6.8 per 1,000 for SVR patients compared with 21.8 per 1,000 for non-SVR patients; a hazard ratio of all-cause mortality of 3.22 for non-SVR patients compared to SVR patients. Taking the estimate from McCombs et al,^22^ this study imposes a hepatitis C death rate which is 3.22-times the natural mortality rate. A graphical example of these two mortality rates is displayed in Figure 3.

The discount rate, *r*, was assumed to be 5%. Utility values, for recovered (*ψ_R_*), infected (*ψ_I_*), and dead (*ψ_D_*) individuals were 1.0, 0.3, and 0.0, respectively.

The model was parameterized in accordance with ledipasvir-sofosbuvir combination therapy, as is recommended by NICE guidelines for treatment of genotype 1 and 4 patients depending on their liver disease stage and treatment history. The recommended course of treatment is 12 weeks for genotype 1 and 4 non-cirrhotic patients who have previously been treated and genotype 1 and 4 patients with compensated cirrhosis. An 8-week treatment course is only recommended for genotype 1 non-cirrhotic and previously untreated patients.^23^ Ledipasvir-sofosbuvir has been observed to have efficacy of 94% in genotype 3 infected patients.^24^ Therefore, efficacies of patented and generic drugs, *e_P_* and *e_G_*, were assumed to be equal at 94%. It would be possible to use the model for other treatments, such as daclatasvir-sofosbuvir, which has so far demonstrated consistently high viral suppression rates (93% to 97%) across genotypes 1–4.^25^

An 8-week treatment course of ledipasvir-sofosbuvir costs £25,986.66, and a 12-week treatment course costs £38,979.99, both without VAT.^26^ The 12-week treatment course cost of £38,979.99 was, therefore, assumed as the patented drug cost, *P_P_*. The cost of a lower-cost future generic was assumed to enter the market at 60% of the patented cost; therefore, a generic of ledipasvir-sofosbuvir would enter at £23,387.99. This was incorporated into the model as the generic cost, *P_G_*. However, a study by Hill et al^27^ on the generic production of hepatitis C drugs found the lowest global price of a 12-week course of ledipasvir-sofosbuvir in 2016 to be USD $507. In another study, Hill et al^28^ examined per-kilogram prices of daclatasvir-sofosbuvir active pharmaceutical ingredients (API) exported from India, suggesting that the cost of generic production of daclatasvir-sofosbuvir combination therapy could be as low as USD $200 for a 12-week course. In 2017, the lowest global price was observed to have reduced to USD $307 for ledipasvir-sofosbuvir and USD $108 for daclatasvir-sofosbuvir.^29^ Therefore, the percentage of the current drug price that generic drugs might enter the market at was a key focus in sensitivity analysis. Many hepatitis C combination therapy drugs are expected to begin to reach patent expiry in 2030.^30^,^31^ The model was run for 20 years, beginning in 2018; therefore, year 12 following model initiation was assumed to see generic introduction. The timing of patent expiry and generic introduction were also subject to sensitivity analysis.

The coverage rates for patented and generic drugs, *a_P_* and *a_G_*, were varied according to the treatment scenario. Two scenarios were compared.

#### Baseline treatment scenario

The first, baseline treatment regimen, *TR_base_*, involved treating patients prior to patent expiry with patented drugs before switching to the generic versions after patent expiry. In this situation, the coverage rates of both drugs, *a_P_* and *a_G_*, were set at 90%.

#### Comparator treatment scenario

In the second, comparator treatment regime, *TR_comp_*, patients were not treated with the patented drug, but instead were treated after patent expiry with generic equivalents. In this second scenario, *a_P_* was set to 0% and *a_G_* was set to 90%.

## Results

### Case study: parameterized model

**Figure 4:**
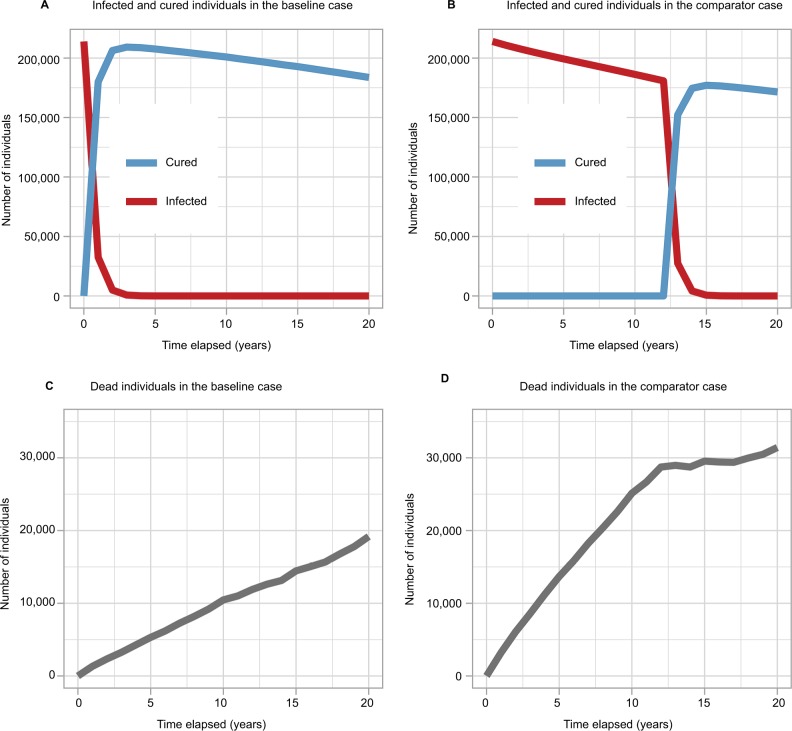
The cumulative number of infected and cured individuals over time in the (**A**) baseline case and (**B**) comparator case. The cumulative number of dead individuals over time in the (**C**) baseline case and (**D**) comparator case.

The number of infected and cured individuals over time for each regimen are displayed in Figures 4A and B. In the comparator case, where patented drugs are not used and generic drug treatment commences only after patent expiry, the number of infected individuals was seen to decline slowly pre-expiry due to mortality. Post-expiry, the number of infected individuals fell and the number of cured individuals rose. However, the comparator case did not cure as many individuals as the baseline case where patients are also offered patented drugs pre-expiry.

The number of deceased individuals over time for both treatment regimens is displayed in Figures 4C, and D. The comparator regimen saw a greater number of deceased individuals overall, driven by deaths in the time period before patent expiry when patients do not receive treatment.

The cost-per-QALY gained for the baseline treatment regimen was £299.88. The cost-per-QALY gained on the comparator treatment regimen was £1,096.47. This resulted in a large negative ICER of –616.65. Therefore, with the parameters outlined in the case study, it was more cost effective to treat patients with more expensive patented drugs pre-patent expiry before switching to generics once they become available, as opposed to waiting for patent expiry to treat patients with cheaper generic drugs.

### Drug costs

**Figure 5:**
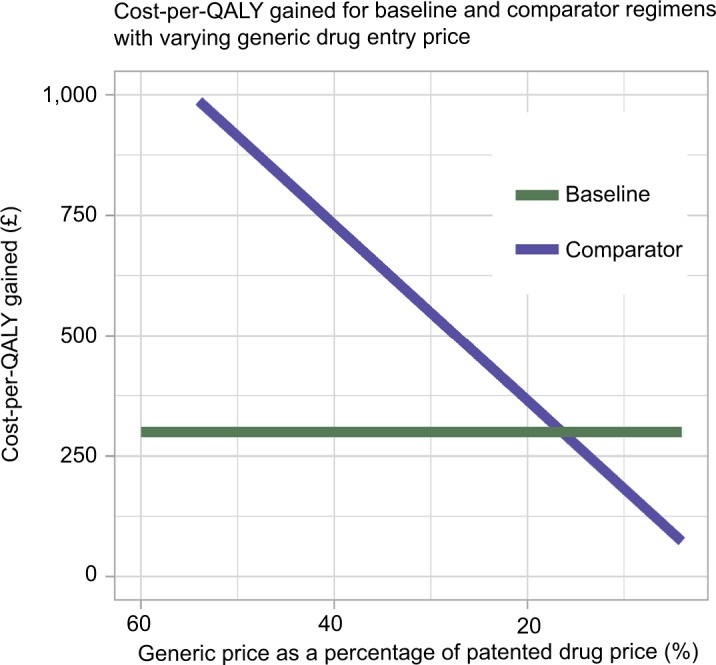
Decrease in the cost-per-QALY gained (£) for the comparator treatment regimen as the price of generic drug at entry (as a percentage of the patented drug price) decreases. **Note:** The cost-per-QALY gained remained approximately constant in the baseline case, because the majority of patients were treated prior to patent expiry. **Abbreviation:** QALY, quality-adjusted life year.

The effect of the cost of generic drugs at market entry upon the cost-per-QALY gained for both treatment regimens was monitored using iterative model runs. Figure 5 shows that decreasing the cost of generic drugs at entry as a percentage of the patented drug cost linearly decreased the cost-per-QALY gained for the comparator treatment regimen. In other words, the lower the cost of the generic drug at entry, the more likely it is to be cost effective to wait until patent expiry to treat patients with generic drugs. On the current model parameterization, the cost-per-QALY gained for the comparator regimen was less than that of the baseline regimen when the cost of the generic drug at entry was <16.40% that of the patented drug. The cost-per-QALY gained was observed to remain approximately constant for the baseline treatment regimen, despite variation in the cost of generic drugs. This occurred because the majority of infected individuals were treated prior to patent expiry in this scenario. Therefore, variation in the cost of generic drugs had minimal impact.

The effect of the patented drug price upon the cost-per-QALY gained for both treatment regimens was also considered. The patented drug price was varied as a percentage of the list price (£38,979.99 for ledipasvir-sofosbuvir). For the baseline regimen, the cost-per-QALY gained was £877.18 at 80%, £548.23 at 50%, and £219.29 at 20%. For the comparator regimen, the cost-per-QALY gained was £239.90 at 80%, £149.94 at 50%, and £59.98 at 20%. Therefore, the comparator regimen remained more cost effective. The baseline treatment regimen became more cost effective when the generic price was <16.40% of the patented drug price, irrespective of the patented drug price.

### Time of generic drug entry

**Figure 6:**
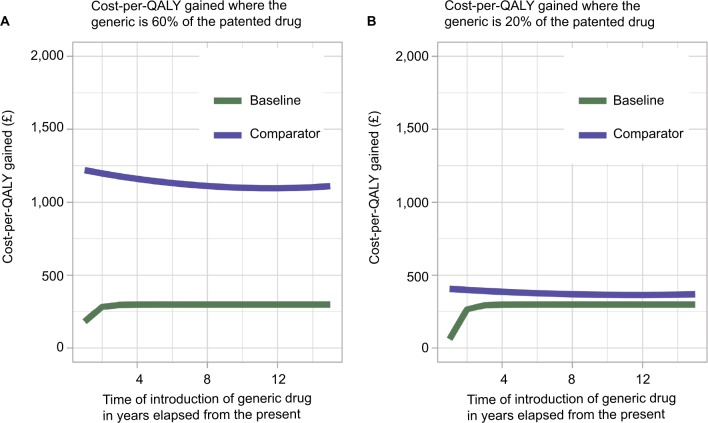
Variation in the cost-per-QALY gained (£) for the baseline and comparator treatment regimens as the time of patent expiry (in years from the present) changes. **Note:** Results are displayed for a scenario where the generic enters the market at (**A**) 60% of the patented drug price and (**B**) 20% of the patented drug price. **Abbreviation:** QALY, quality-adjusted life year.

The cost-per-QALY gained was lowest for the baseline case if the patent expired within approximately 2 years from the present. It then plateaued, and the time of generic drug entry had no further notable effect on the cost-per-QALY gained (see Figure 6). It was hypothesized that this was a consequence of the study population being closed. As the model moves further away from the present, individuals are either treated or die on the baseline case. The comparator treatment regimen had a steady cost-per-QALY gained, irrespective of the time of generic drug market entry. However, the cost-per-QALY gained for the comparator scenario was greatly reduced if the cost of the generic drug was reduced, although still largely unaffected by the time of generic entry (see Figure 6B).

### Utility values

In the parameterized model, the utility value of infected individuals, ψ_I_, was set to 0.3. The utility value was varied to investigate its effect upon the cost-per-QALY gained. This was an important parameter to investigate as the assignment of utility values may be vulnerable to a level of subjectivity. Increasing the utility value of infected individuals from 0.3 linearly decreased the cost-per-QALY gained for both the comparator and baseline treatment regimens. However, a change in utility value did not make the comparator regimen more cost effective than the baseline regimen. The costs-per-QALY gained with a utility value of 0.3 for infected individuals were £299.88 and £1,096.47 for baseline and comparator regimens, respectively. For a utility value of 0.9, these reduced to £289.00 and £636.51, respectively. For a utility value of 0.1, they increased to £303.68 and £1,444.25, respectively.

### Different regimens: treatment of a high-risk subpopulation

Patients with certain manifestations of hepatitis C are thought to have a higher mortality risk. These include cirrhotic patients or patients with a particular hepatitis C genotype. Therefore, the model was extended to consider an alternative comparator treatment regimen where only high-risk patients were treated with patented drugs prior to patent expiry. Following patent expiry, all patients were treated with generic drugs. A high-risk subpopulation was defined as 10% of the infected population, equally sampled across age groups. The high-risk population had a mortality rate 10-times that of the natural mortality rate. Therefore, the high-risk infected population was defined as

$$HR_{\left(a,t\right)}=\left\{ \begin{array}{l} \left(HR_{\left(a-1,t-1\right)}-\alpha_{P_{HR}}\cdot e_{P}\cdot HR_{\left(a-1.t-1\right)}\right)\cdot \left(1-\mu_{HR_{\left(a\right)}}\right)if\;t<T^{*}\\ \left(HR_{\left(a-1,t-1\right)}-\alpha_{G_{HR}}\cdot e_{G}\cdot HR_{\left(a-1.t-1\right)}\right)\cdot \left(1-\mu_{HR_{\left(a\right)}}\right)if\;t\geq T^{*} \end{array}\right.$$ (17)

and the total number of infected individuals over the time horizon, *T*, was calculated as the sum of the total number of infected (normal risk) and high-risk individuals. The utility value for high-risk individuals was set at 0.1. The baseline treatment scenario was kept as defined in the “Baseline treatment scenario section”. In the high-risk comparator scenario, non-high-risk infected individuals were not treated prior to patent expiry. High-risk individuals were treated at a rate of 90% prior to patent expiry. All patients were treated after patent expiry with generic drugs at a rate of 90%. All other parameters were unchanged.

The cost-per-QALY gained in the baseline scenario with a high-risk group was £299.49. The cost-per-QALY gained in the comparator scenario with a high-risk group was £961.29, resulting in a large negative ICER of –616.65. The reduction in the cost-per-QALY gained for the comparator scenario when a high-risk group is not considered is, therefore, £135.18 (see the Drug costs section). However, treatment of a high-risk group at an earlier stage and other infected individuals at a later stage was not more cost effective than treating all individuals at an early stage with high-cost, patented drugs, irrespective of their mortality risk.

### Open population

The effect of having an open as opposed to a closed population upon the cost-per-QALY gained was investigated. Consideration of an open population was thought to be important because a closed population may skew cost-effectiveness estimates. As individuals are cured or die, few patients may be left to treat at later stages after patent expiry in a closed population.

Since the majority of individuals exhibit no symptoms when they become infected, there is little information about the incidence of hepatitis C and, therefore, the number of new cases per year in the UK.^32^ Therefore, the number of new cases each year was varied to investigate the effect on cost-per-QALY gained. The number of new cases per year was calculated as a proportion of the original infected population, *ρ*, distributed across age groups in accordance with the distribution derived from Mohsen (2001)^19^ (see the case study section and Figure 2). In this sense, the open population represents new diagnoses of existing cases rather than newly acquired infections. This approach was adopted because hepatitis C can often go many years without detection. This proportion was varied between 0.1% and 25%. This yielded new cases per year varying between ~214 and ~52,642. Equation (9) was modified to

$$I_{\left(a,t\right)}=\left\{ \begin{array}{l} \left(I_{\left(a-1,t-1\right)}-\alpha_{P}\cdot e_{P}I_{\left(a-1.t-1\right)}\right)\cdot \left(1-\mu_{I_{\left(a\right)}}\right)+\rho \cdot I_{\left(a,0\right)}\;if\;t<T^{*}\\ \left(I_{\left(a-1,t-1\right)}-\alpha_{G}\cdot e_{G}I_{\left(a-1.t-1\right)}\right)\cdot \left(1-\mu_{I_{\left(a\right)}}\right)+\rho \cdot I_{\left(a,0\right)}\;if\;t\geq T^{*} \end{array}\right.$$ (18)

**Figure 7:**
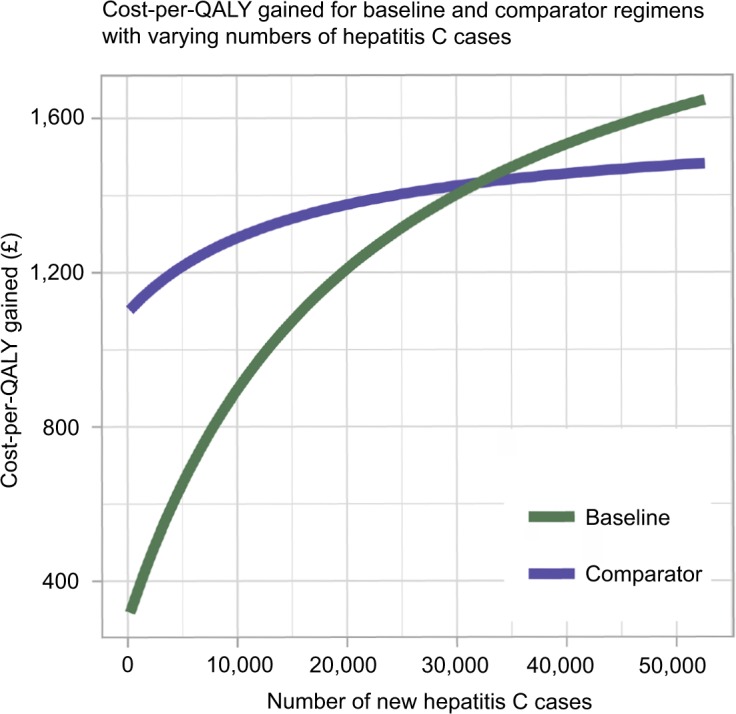
Variation in the cost-per-QALY gained (£) for the baseline and comparator treatment regimens as the number of new hepatitis C cases per year changes. **Abbreviation:** QALY, quality-adjusted life year.

Variation in the cost-per-QALY gained as a result of increasing the number of new cases per year is displayed in Figure 7. The baseline treatment regimen was found to be more cost effective until the number of new cases per year became very high, at which point the comparator treatment regimen became more cost effective. However, the number of new cases per year needed to trigger this scenario was very high; the comparator treatment regimen only became more cost effective when there were more than ~32,000 new cases per year. With the number of people currently living with hepatitis C in the UK was estimated to be ~214,000, it is unlikely that this number of new cases would present in a year in the UK.

## Discussion

The mathematical framework in this study has been applied to hepatitis C as a test case comparing two treatment scenarios: a baseline case where all individuals are treated with patented drugs before switching to generic drugs after patent expiry; a comparator case where no-one is treated until after patent expiry with generic drugs.

Cost is a limiting factor in hepatitis C treatment. A course of ledipasvir-sofosbuvir costs as much as £38,979.99 for 12 weeks, excluding VAT.^25^ Despite this, this study has suggested that it is more cost effective in terms of the cost-per-QALY gained to treat patients at this high price rather than wait until generic release. Varying the cost of the generic drug at market entry suggested that this is true if the generic enters at prices down to a threshold of 16.40% of the patented cost. A meta-analysis by Simmons et al^15^ emphasizes that an SVR is achievable even in high-risk hepatitis C populations. Using the presented model, restricting treatment using high-cost patented drugs to a high-risk infected group was not more cost effective than treating everyone with the same drugs. Therefore, this study supports the conclusion of Simmons et al^15^ and, in addition, suggests not only that all patients should be treated, but that patients should be treated sooner rather than later in order to be cost effective, even if the price for current treatment is significantly higher than what it might be in the future.

Hill et al^29^ have reported that the lowest current global price for ledipasvir-sofosbuvir is USD $307. This is significantly less than 16.40% of the patented cost of ledipasvir-sofosbuvir (£6,392.72). If generic drugs entered the market at $307, then late treatment with generic drugs would be more cost effective than early treatment with patented drugs.

However, I present two arguments that the possibility of very low-cost future generic drugs should not override treatment in the present with higher cost drugs. First, the fact that ledipasvir-sofosbuvir can be produced for USD $307 does not mean that they will necessarily enter the UK market at this price. Second, the cost-effectiveness of late treatment with generic drugs is sensitive to other parameters such as the timing of patent expiration. The model showed that, if patent expiry is very soon in the future then it is almost always substantially more cost effective to treat patients now with high-cost drugs. The cost-per-QALY gained was as low as £182.21 if patent expiry was only 1 year in the future. Late patent expiry might make it more likely that a very low-cost generic drug could make late treatment cost effective. However, late patent expiry also introduces more uncertainty; it is more difficult to predict what the generic entry price might be or whether existing drug companies will make efforts to prevent uncontrolled generic release. Due to the large difference in cost-effectiveness between the two treatment regimens analyzed in this study (£299.88 compared with £1,096.47 per QALY gained for baseline and comparator, respectively), an error due to uncertainty could be costly.

Crucially, the low price of USD $307 is not a hypothetical future generic cost, but a real cost in the present. Combination therapies including ledipasvir-sofosbuvir are already being produced in other countries at prices exceedingly lower than the price for the same therapy in the UK. This study demonstrates the cost-effectiveness of treating patients with high-cost patented drugs. This should not distract from the fact that health care costs could be reduced colossally if the price of hepatitis C treatment was reduced.

A middle ground option may be negotiation of lower-cost patented treatments in the present so that patients can be treated in the immediate future more cost-effectively. In 2016, the Australian government negotiated with pharmaceutical companies to secure a commitment of AUD $1 billion over 5 years to ensure major discounts on drug prices. A maximum cap was placed upon expenditure each year, but no cap upon the number of people who could be treated.^33^ Approximately 230,000 people are thought to be living with chronic hepatitis C in Australia; therefore, if all infected people are treated during the 5-year negotiation period then the cost of treatment would be approximately AUD $4,350 per person (equivalent to USD ~$3,375, ~£2,370 in April 2018) compared to £38,979.99 for a 12-week course of ledipasvir-sofosbuvir in the UK.^26^,^34^ The model found that if the generic price was £2,370 in the UK then it would be more cost effective to treat now at that price than wait for generics, assuming that generics would be introduced at 60% of this price. The cost-per-QALY gained was only £18.23 for the comparator regimen and £66.67 for the baseline regimen with an ICER of −37.49. This is reduction of £281.65 per-QALY gained for the comparator regimen compared with a situation where the patented drug price is £38,979.99 as in the UK. Price negotiation with pharmaceutical companies may be a crucial step in providing access to drugs.

Further extensions of the mathematical framework presented in this study could consider the duration that an infected individual has had the disease. As well as having an increased risk of mortality due to aging, individuals may have a higher risk of mortality as a function of the duration of time they have been infected. Therefore, neglecting this element may even underestimate the cost-per-QALY gained for treatment regimens postponing treatment until after patent expiry. It should be noted that screening for early infection is difficult in the case of hepatitis C.

This study has presented a mathematical framework which is capable of comparing the cost-effectiveness of different treatment regimens considering future low-cost generic drugs. The model has been applied to hepatitis C as a test case, although the framework can be adapted for a wide range of diseases. Results have demonstrated that, despite the very high cost of patented hepatitis C drugs and the significantly reduced cost of their generic counterparts, it is almost always more cost effective in terms of the cost-per-QALY gained to treat now using high price drugs. The results of this study have shown that it is only more cost effective to postpone treatment until after patent expiry under very specific conditions; however, I have argued that, even under these conditions, there is a high degree of uncertainty as to whether the parameters necessary for the cost-effectiveness of postponed treatment will hold in the future. Negotiation of patented drug prices with pharmaceutical companies may be a crucial step in cost effective treatment of hepatitis C.
